# Multi-Omics Analyses Unveil the Effects of a Long-Term High-Salt, High-Fat, and High-Fructose Diet on Rats

**DOI:** 10.3390/foods15010171

**Published:** 2026-01-04

**Authors:** Yue Yao, Xiao Wu, Hao Wu, Weiwei Su, Peibo Li

**Affiliations:** Guangdong Engineering and Technology Research Center for Quality and Efficacy Re-Evaluation of Post-Marketed TCM, Guangdong Provincial Key Laboratory of Plant Stress Biology, State Key Laboratory of Biocontrol, School of Life Sciences, Sun Yat-sen University, Guangzhou 510275, China; yaoy77@mail2.sysu.edu.cn (Y.Y.); wuxiao53@mail2.sysu.edu.cn (X.W.); wuhao8@mail.sysu.edu.cn (H.W.); lsssww@mail.sysu.edu.cn (W.S.)

**Keywords:** high-salt high-fat and high-fructose diet, brain damage, proteomics, serum metabolome, gut microbiome

## Abstract

Background: Unhealthy diets characterized by high salt, fat, and fructose content are established risk factors for metabolic and cardiovascular disorders and may have indirect effects on cognitive function. However, the combined impact of a high-salt, high-fat, and high-fructose diet (HSHFHFD) on systemic physiology and brain health remains to be fully elucidated. Methods: Sprague-Dawley (SD) rats received a customized high-salt, high-fat diet supplemented with 30% fructose water for 18 weeks. Physiological and brain parameters were assessed, in combination with multi-omics analyses including brain proteomics and metabolomics, serum metabolomics, and gut microbiota profiling. Results: HSHFHFD significantly elevated blood glucose, blood pressure, and serum levels of TG, TC, and LDL in rats. Serum metabolomic profiling identified over 100 differentially abundant metabolites in the Model group. Proteomics, metabolomics, and gut microbiome integration revealed pronounced alterations in both brain proteomic and metabolomic profiles, with 155 differentially expressed proteins associated with glial cell proliferation and 65 differential metabolites linked to fatty acid and amino acid metabolism, among others. Experimental validation confirmed marked upregulation of GFAP and Bax protein, concomitant with downregulation of ZO-1 and occludin. Furthermore, HSHFHFD perturbed the CREB signaling pathway, leading to diminished BDNF expression. The levels of inflammatory factors, including IL-6, IL-10, IL-1β and TNFα, were significantly elevated in the brain. Oxidative stress was evident, as indicated by elevated malondialdehyde (MDA) levels, increased superoxide dismutase (SOD) activity, and altered NAD^+^/NADH ratio. Additionally, HSHFHFD significantly reduced the abundance of beneficial gut bacteria, including *Lactobacillus*, *Romboutsia*, and *Monoglobus*. Conclusions: HSHFHFD-induced depletion of gut *Lactobacillus* spp. may disrupt the linoleic acid metabolic pathway and gut–brain axis homeostasis, leading to the impairment of neuroprotective function, blood–brain barrier dysfunction, and exacerbated neuroinflammation and oxidative stress in the brain. These effects potentially increase the susceptibility of rats to neurodegenerative disorders.

## 1. Introduction

A balanced diet is essential for maintaining optimal health. However, the global proliferation of high-salt/fat/sugar diets poses escalating health threats. The global average daily sodium intake for adults is 4310 mg, equivalent to 10.78 g of salt, which far exceeds the WHO recommendation of less than 2000 mg of sodium or less than 5 g/d of salt [1]. A high-salt diet primarily contributes to hypertension and increases the risk of cardiovascular disease, stomach cancer, and obesity, with approximately 1.89 million deaths annually attributed to excessive sodium intake [2]. High-fat diets exhibit a well-established etiological association with obesity, a pandemic affecting over 1 billion individuals globally as of 2022 [3]. Moreover, obesity resulting from high-fat diets also heightens the risk of cardiovascular diseases such as atherosclerosis and myocardial infarction. Statistics indicate that the prevalence of moderate to severe atherosclerosis among obese women stands at 52%, compared to 28% among women with non-central obesity [4]. Similarly, excessive sugar consumption constitutes a major risk factor for obesity, diabetes mellitus, coronary heart disease and hypertension [5], evidenced by urban populations deriving 42.1% of free sugars from sweetened and dairy-based beverages [6].

Metabolic disorders induced by high-salt/fat/sugar diet may foster the onset and progression of metabolic, cardiovascular, and cerebrovascular conditions [7]. Excessive salt intake represents a major dietary contributor to elevated blood pressure, thereby increasing the risk of cardiovascular and cerebrovascular diseases [8]. Concurrently, high-fat diets impair transporter proteins within the blood–brain barrier (BBB), compromising fructose delivery for cerebral energy metabolism and subsequently affecting the functions of the cerebral cortex and hippocampus [9]. This can manifest as delayed responses, decreased memory, and attentional difficulties [10]. Moreover, high-fat diets are implicated in the pathogenesis of cognitive disorders, notably Alzheimer’s disease (AD) [11]. Rodent studies demonstrate that even minimal intake of high-fat food over short durations at any life stage can impair performance in spatial learning and memory tasks, such as the Morris water maze and Barnes maze [12]. High-sugar diets are linked to neurological impairments, including anxiety, depression, and fear-related behaviors, alongside perturbations in molecular and neurochemical expressions [13]. Overconsumption of fructose can result in neuroinflammation, excessive activation of microglial cells, and oxidative stress within the nervous system [14]. Furthermore, high-concentration fructose or sugar administration in rodents can lead to cognitive and memory deficits [15].

Dietary composition also affects the gut microbiota. The human gastrointestinal tract harbors over 10^14^ bacteria, a number estimated to be 10 to 100 times greater than that of host somatic cells. The gut microbiota function as an endocrine organ, producing metabolites that critically regulate the host immune system and metabolic functions [16]. There is also a bidirectional regulation between the gut microbiota and the brain, known as the ‘gut–brain axis,’ wherein microbial metabolites regulate physiological brain functions via neurohumoral pathways [17]. Short-chain fatty acids (SCAFs), metabolites of the gut microbiota, are associated with various neurological disorders. Research by Moira et al. [18] suggests that lipopolysaccharides (LPS) derived from the gut microbiota, alongside SCFAs such as acetate, valerate, and butyrate, correlate with cerebral amyloid deposition, a process potentially mediated by inflammation and endothelial dysfunction. Colombo et al. [19] further demonstrated that SCFAs serve as pivotal mediators along the gut–brain axis, potentially promoting Aβ deposition by modulating microglial phenotypes. Moreover, SCFAs influence the progression of AD and Parkinson’s disease (PD) through epigenetic regulation and immunomodulation pathways [20].

Dietary composition profoundly modulates gut microbiota, inducing significant shifts in microbial abundance, diversity, and community structure. Research indicates that a high-salt diet can alter microbiota by increasing the *Firmicutes*/*Bacteroides* ratio, thereby affecting the production of SCFAs [21]. A high-fat diet significantly depletes *Bifidobacterium* abundance in the gut microbiota of mice [22]. Consumption of a high-fructose diet leads to an increase in the *Firmicutes*/*Bacteroides* ratio, and an increase in the proportion of *Proteobacteria*, which is one of the main sources of LPS [23,24]. Alterations in the gut microbiota may lead to changes in its metabolic byproducts, such as SCAFs. These metabolites influence brain function via the vagus nerve or the bloodstream, potentially causing cognitive impairment and emotional dysregulation [25]. Guo et al. [26] confirmed that a combined high salt and high fat diet induces dysbiosis, damages the gut, alters neurotransmitter metabolism in both the intestine and brain, and consequently leads to changes in brain function and circulating RNA profiles. A high-fat diet disrupts the serotonin (5-HT) signaling system, affecting neurotransmission in both the gut and brain [27]. Moreover, dysbiosis of the gut microbiota may also precipitate cerebral inflammation and compromise the blood–brain barrier. For instance, high-fat diets may disrupt the integrity of the blood–brain barrier via oxidative stress and inflammatory pathways (such as TLR4/MyD88/NF-κB), thereby facilitating the entry of peripheral inflammatory mediators into the brain and exacerbating neuroinflammation [28].

In China, the per capita intake of salt, fat, and sugar has surpassed WHO recommendations, significantly contributing to the rising incidence of CVD in the country. In response, the Healthy China Initiative urges society to mitigate the risk of CVD by reducing the consumption of salt, oil, and sugar. While extensive literature exists on the individual effects of diets high in salt, fat, or fructose, the specific combined effects of HSHFHFD remain underexplored. Therefore, the present study aimed to investigate the effects of these combined dietary components on the brain and body of Sprague-Dawley (SD) rats. To this end, a comprehensive array of physiological indicators was assessed, including blood glucose and systolic blood pressure, and markers of oxidative stress and inflammatory responses. Subsequent multi-omics analyses encompassed brain proteomics and metabolomics, serum metabolomics, and gut microbiota profiling. The results indicated that HSHFHFD adversely impacted the gut microbiota, particularly *Lactobacillus* spp., which was associated with dysregulation of the linoleic acid metabolic pathway. These alterations were closely related to brain health, which may affect the neuroprotective function of brain and heighten metabolic and neurodegenerative diseases in rats.

## 2. Materials and Methods

### 2.1. Animals and Treatment

Eight weeks Specific Pathogen-Free (SPF) male Sprague-dawley (SD) rats (180 ± 20 g body weight) were obtained from the Laboratory Animal Center of Sun Yat-sen University and housed in the barrier system of Sun Yat-sen University Laboratory Animal Center. The rearing conditions were as follows: temperature 20~26 °C, relative humidity 50%~60%, 12 h day and night alternation. All experimental procedures received prior approval from the IACUC of Sun Yat-sen University (Approval No. SYSU-IACUC-2022-001964) and were performed in strict compliance with the institutional guidelines.

The rats were acclimatized for 7 days and randomly divided into two groups of 10 rats each: the Blank group and the Model group. Rats in the Blank group received a standard diet and water. The Model group received a high salt, high fat diet, which includes 20% lard, 5% egg York, 10% salt, and 2% cholesterol. And a high-sugar diet was constituted by providing 30% fructose water. During the experiment, the rats in all groups had free access to food and water. General health status and behavior were monitored daily. To minimize unpredictable effects on animal condition from procedures requiring anesthesia and blood sampling for certain parameter measurements, blood glucose was assessed every 6 weeks, blood pressure every 4 weeks, and serum levels of TG, TC, LDL, HDL, TP, ALB, ALT, AST, and UREA were measured at 9 and 18 weeks. At the end of the modeling period, the rats were fasted for 12 h then serum samples, fecal matter, and brain tissues were collected for subsequent analysis.

### 2.2. Oxidative Stress Indicators Measurement

The whole brain tissue samples were collected and homogenized on ice in an appropriate extraction buffer. After centrifugation, the supernatant was collected, and NAD^+^/NADH (Beyotime, Shanghai, China), superoxide dismutase (SOD, Beyotime, Shanghai, China), and malondialdehyde (MDA, dojindo, Shanghai, China) were detected using commercially available assay kits.

### 2.3. Enzyme-Linked Immunosorbent Assay (ELISA)

The levels of TNF-α, interleukin (IL)-6, interleukin (IL)-10, and interleukin (IL)-1β in brain were determined using ELISA. Whole brain tissue samples were homogenized in PBS and centrifuged at 3000 rpm for 10 min. The resulting supernatant was collected, and cytokine concentrations were quantified using commercial ELISA kits (Invitrogen, Carlsbad, CA, USA).

### 2.4. Western Blot

To investigate the effects of HSHFHFD on protein expressions in the brain, Western blot analysis was performed. Brain tissue samples were lysed using a RIPA buffer (Beyotime, Shanghai, China). The BCA assay (Beyotime, Shanghai, China) was used to quantify the protein concentration of the lysis supernatant after centrifuging at 12,000× *g* for 30 min. Then, the normalized proteins were mixed with SDS-PAGE sample loading buffer and heated at 100 °C for 10 min. The samples were then separated by 12% SDS-PAGE and transferred to polyvinylidene fluoride (PVDF) membranes. The PVDF membranes were incubated with a blocking solution containing 5% non-fat dry milk for 2 h at room temperature. After blocking, the membranes were incubated overnight at 4 °C with various antibodies. These included glial fibrillary acidic protein (GFAP, 1:1000 dilution, Invitrogen, Carlsbad, CA, USA), zonula occludens-1 (ZO-1, 1:100 dilution, Invitrogen, CA, USA), Occludin (Abcam, 1:1000 dilution, Cambridge, UK), CREB (cyclic-AMP response binding protein, CST, 1:1000 dilution, Danvers, MA, USA), *p*-CREB (CST, 1:1000 dilution, Danvers, MA, USA), brain-derived neurotrophic factor (BDNF, 1:20,000 dilution, Abclonal, Wuhan, China), Bcl-2 (Abcam, 1:1000 dilution, Cambridge, UK), Bax (Bcl-2-associated X protein, CST, 1:1000 dilution, Danvers, MA, USA), and Vinculin (Abcam, 1:10,000 dilution, Cambridge, UK). The membranes were then washed five times with TBST and incubated with corresponding HRP-conjugated secondary antibodies for 1 h at room temperature. Finally, protein bands were visualized using a chemiluminescence imaging system (Tanon, Shanghai, China), and the protein levels were quantified using the ImageJ software (Version 1.53t).

### 2.5. LC-MS/MS-Based Quantitative Proteomic Profiling of Brain Tissue

The brain tissue samples were homogenized in 0.5 mL RIPA buffer for 4 min and subsequently sonicated on ice for 2 min. After centrifugation of the lysate (12,000 rpm, 10 min, 4 °C), the supernatant was collected. The total protein in the supernatant was quantified using the BCA assay. The distilled water was added into 100 mg protein extracts of each sample at a final concentration of 1 mg/mL. The sample was precipitated by adding five volumes of pre-chilled acetone (−20 °C), vortexing thoroughly, and incubating overnight at −20 °C. Precipitates were recovered by centrifugation (12,000 rpm, 10 min, 4 °C), followed by supernatant removal. Following incubation with 5 mL of 200 mM dithiothreitol for 1 h at 55 °C, samples were exposed to 10 mM iodoacetamide and incubated at room temperature for 15 min. The digestion of the sample was conducted overnight with trypsin (Promega, Madison, WI, USA) at a 50:1 mass ratio. After TMT labeling, the resulting peptides were desalted using C18 disks (Sigma-Aldrich, St. Louis, MO, USA), vacuum-dried, and separated using Waters XBridge BEH C18 XP Column (150 mm × 2.1 mm, Waters Corporation, Milford, MA, USA) to obtain high PH fractionation peptides. Each fraction was further separated using a reversed-phase column (Reprosil-Pur 120 C18-AQ, Dr. Maisch GmbH, Remseck, Germany) on a nanoUPLC system (EASYnLC 1200, Thermo Fisher Scientific, Waltham, MA, USA). The mass spectral analysis was performed using a Q Exactive HFX Orbitrap instrument (Thermo Fisher Scientific, Waltham, MA, USA) equipped with a nanoelectrospray ion source. The gradient elution employed solvents A (0.1% formic acid in 80% acetonitrile) and B (0.1% formic acid in water) as follows: 0–2 min, 2 to 5% A; 2–70 min, 5% to 22% A; 70–86 min, 22% to 45%A; 86–88 min, 95% A.

Protein identification and quantification were performed using Proteome Discoverer software (version 2.4.1.15, Thermo Fisher Scientific). In the protein sequence database, uniprot-Rattus+norvegicus-10116-2020-10. Fasta was selected, and the Sequest HT search engine was used to search the database. Partial least squares-discriminant analysis (PLS-DA) was applied for dimensionality reduction and pattern recognition within each experimental group. The differentially expressed proteins were determined by screening the proteins with VIP value > 1 and *p* < 0.05 that met the orthogonal partial least squares-discriminant analysis (OPLS-DA) between groups. The protein–protein interaction network (PPI) of differentially expressed proteins was analyzed using the STRING database (https://string-db.org/, accessed on 5 February 2024). DAVID online tools (https://davidbioinformatics.nih.gov/, accessed on 7 February 2024) were applied to analyze differentially expressed proteins in a biological process and KEGG pathway enrichment analysis.

### 2.6. 16S rRNA Gene Sequencing and Bioinformatic Analysis

Fresh fecal samples were collected from each rat at the experimental endpoint, and the fecal DNA was extracted using the PowerSoil DNA Extraction Kit (MO BIO Laboratories, Inc., Carlsbad, CA, USA). DNA purity and concentration were assessed with a Nanodrop ND-1000 spectrophotometer (Thermo Electron Corporation, Waltham, MA, USA). The V3-V4 hypervariable regions of the bacterial 16S rRNA gene were then amplified by PCR using region-specific primers. Final PCR products were purified from unincorporated nucleotides and primers using the QIAquick PCR Purification Kit (Qiagen, Valencia, CA, USA). Purified samples were normalized to equal DNA concentration and sequenced using the Illumina MiSeq sequencer (Illumina, San Diego, CA, USA). Alpha diversity of the gut microbiota was analyzed using Shannon, Pielou, and phylogenetic diversity (PD) whole tree metric. Differences in alpha diversity indices between groups were compared using the Wilcoxon rank-sum test. Principal component analysis (PCA) and principal co-ordinates analysis (PCoA) were applied to evaluate the beta diversity of each group based on the Bray distances; PERMANOVA was used to test the compositional differences between groups. Linear discriminant analysis effect size (LEfSe) was used to analyze the differences between groups. The bacterial differences between the two groups were tested by Welch’s *t*-test at the genus level. Phylogenetic investigation of communities by reconstruction of unobserved states (PICRUSt) was employed to analyze community function, and Welch’s *t*-test was used to test between the two groups.

### 2.7. Metabolomics Analysis of Brain Tissue and Serum

Brain tissue samples were prepared for untargeted metabolomic analysis by homogenization in ice-cold methanol/acetonitrile (1:1, *v*/*v*; 400 µL) containing deuterated coumaric acid (20 µg/mL) as an internal standard (IS). Following homogenization, samples were incubated at −20 °C for 1 h, after which 50 µL were taken from each sample to prepare quality control (QC) samples. The samples were centrifuged at 4 °C for 20 min at 15,000 g. Then, 400 µL of the supernatant was collected and evaporated using a liquid nitrogen evaporator at 37 °C under a stream of nitrogen gas. The residue was reconstituted with 100 µL of methanol, centrifuged at 4 °C for 10 min at 15,000× *g*, and the supernatant was transferred to a vial for sample analysis.

Serum metabolomic sample preparation was initiated by adding 400 μL of ice-cold methanol/acetonitrile (1:1, *v*/*v*; containing 20 μg/mL myristic acid-D27 IS) to 100 μL of thawed serum. Proteins were precipitated by vortexing for 30 s and incubating at −20 °C for 1 h. The supernatant (200 μL collected after centrifugation at 15,000× *g* for 20 min) was used for analysis, with an injection volume of 10 μL. QC samples were prepared by mixing equal volume (5 μL) of each sample. The pooled QC samples were injected every eight samples during detection to monitor system performance and maintain stability.

Untargeted metabolomics analysis was performed using UFLC-XR (Shimadzu Corp, Kyoto, Japan) coupled to a hybrid triple quadrupole time-of-flight mass spectrometer (Triple TOF™ 7600^+^, AB Sciex, Forster City, CA, USA) equipped with an electrospray ionization source. The chromatographic separation was performed on an ACQUITY UPLC^®^ HSS T3 column (2.1 × 100 mm, 1.8 μm; Waters, Milford, CT, USA) at 50 °C with a flow rate of 0.3 mL/min. The elution gradient program with solvent A (deionized water with 0.1% formic acid) and solvent B (acetonitrile with 0.1% formic acid) was set as follows: 0–1.5 min, isocratic 1% B; 1.5–13 min linear gradient from 1 to 99% B; 13–16.5 min isocratic 99% B, and then back to 1% B in 3.5 min. The instrumental settings of the Q-TOF-MS/MS were set as recommended by AB Sciex: ion source gas 1 and gas 2 were both 55 psi, curtain gas was 35 psi, ion source temperature was 550 °C, ion spray voltage floating was 5500 V in positive mode while 4500 V in negative mode, collision energy was 30 V, collision energy spread was 15 V, and declustering potential was 80 V. Nitrogen was used as nebulizer and auxiliary gas. Serum samples were analyzed in both positive and negative ionization modes with a scan mas to charge (m/z) range of 50 to 1500 Da. Data were collected using Analyst^®^ software (Version 1.7.2, AB Sciex, Foster City, CA, USA) in information-dependent acquisition mode.

The peak table acquisition software of the One-MAP metabolic cloud platform (http://www.5omics.com/, accessed on 2 March 2024) was used to convert the collected raw data into mzML format, and the primary and secondary mass spectra were extracted. The obtained peak list was uploaded into the MetDNA2 platform (http://metdna.zhulab.cn/, accessed on 10 March 2024), and the secondary mass spectrometry data obtained above were uploaded at the same time for qualitative identification analysis of metabolic mass spectrometry characteristics. In addition, the peak table will be imported into MetaboAnalyst (http://www.metaboanalyst.ca/, accessed on 10 March 2024) for peak filling missing values, before the stoichiometric analysis, the data will be processed by normalizing with the internal standard intensity. The obtained data matrix was imported into SIMCA-P software (version 14.1) for PLS-DA dimensionality reduction analysis. Metabolites with VIP value > 1 and *p* < 0.05 of OPLS-DA were selected as characteristic differential metabolites. Comprehensive metabolomic data analysis, including data preprocessing and pathway enrichment analysis, was performed using MetaboAnalyst and the KEGG database.

### 2.8. Statistical Analysis

SPSS software (Version 27.0) and GraphPad Prism (version 8.0.2, La Jolla, CA, USA) software were used for data analysis and image rendering. Student’s *t*-test was used to compare the data between the two groups. *p* < 0.05 was considered significant. The difference was considered to be extremely significant when *p* < 0.01. The results are presented as mean ± standard deviation ($\bar{x}$ ± s).

## 3. Results

### 3.1. Effects of HSHFHFD on Blood Glucose, Systolic Blood Pressure, and Serum Biochemical Indices and Metabolomics in Rats

**Figure 1:**
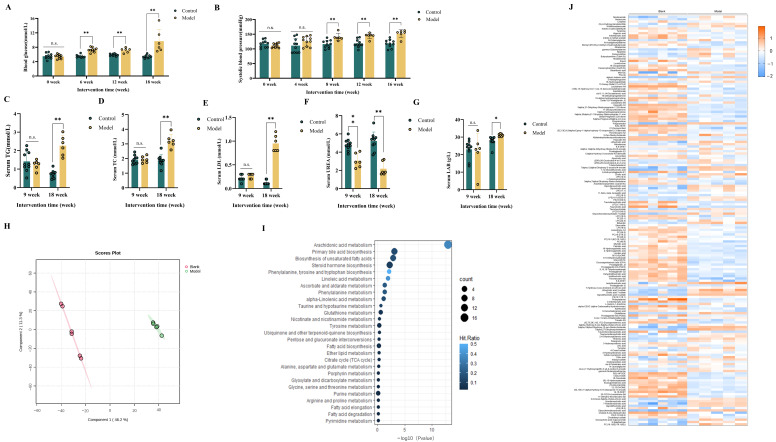
Blood glucose/pressure levels, the serum biochemical parameters, and serum metabolomics analysis of rats. (**A**) Blood glucose levels of rats during modeling; (**B**) Systolic blood pressure values of rats during modeling; (**C**) Serum total triglyceride levels in each group of rats; (**D**) Serum total cholesterol levels in each group of rats; (**E**) Serum total Low-density lipoprotein levels in each group of rats; (**F**) Serum total UREA levels in each group of rats; (**G**) Serum total albumin levels in each group of rats; (**H**) PLS-DA score plot for the Blank group and Model group; (**I**) Heat map of differential metabolites between Blank and Model groups; (**J**) Biological process analysis of the differential metabolites between the Blank group and Model group; Data are expressed as $\bar{x}$ ± *s* (*n* = 6~10); n.s.: no significant difference; * *p* < 0.05; ** *p* < 0.01.

To investigate the impact of HSHFHFD on blood glucose and systolic blood pressure in rats, we conducted regular measurements of these parameters throughout the study as the methods described. After six weeks of dietary intervention, the Model group showed a significant increase in blood glucose compared to the Blank group (*p* < 0.01, Figure 1A). Hyperglycemia persisted until the end of the experiment, suggesting that the HSHFHFD had a substantial effect on glucose regulation in the Model animals. Similarly, the systolic blood pressure of the rats was significantly higher in the Model group than in Blanks after eight weeks of dietary modeling (*p* < 0.01, Figure 1B). The increase in blood pressure remained consistent until the end of the study, indicating that the diet significantly increased blood pressure in the rats. Collectively, these findings demonstrate that HSHFHFD significantly and persistently disrupts both glycemic control and blood pressure regulation in the experimental model.

To further investigate the effects of a HSHFHFD, serum was analyzed at the end of the modeling period. Serum analysis revealed significantly elevated levels of three key lipid metabolism indices (e.g., TC, TG, LDL-C) in the Model group compared to the Blank group (*p* < 0.01, Figure 1C–E), indicating pronounced dyslipidemia in HSHFHFD-fed rats. This metabolic disturbance is associated with an increased risk of cardiovascular and cerebrovascular diseases. Additionally, serum analysis revealed a decrease in urea (*p* < 0.01) and an increase in albumin (*p* < 0.05) in the model rats compared to the blank rats. These findings imply potential impairment of liver function in the Model group.

Given the observed alterations of serum biochemical indices suggesting metabolic disorder risk in the Model group, serum non-targeted metabolomics was employed to comprehensively characterize the impact of the HSHFHFD on systemic metabolism. Serum metabolomes were profiled using liquid chromatography-mass spectrometry in both positive and negative ion modes. The results of the PLS-DA dimensionality reduction analysis of the two groups are shown in Figure 1H, which showed that there was a significant separation between the Blank group and the Model group, indicating that the serum metabolic phenotypes of the rats on the HSHFHFD appeared to be significantly different from those of the healthy blank rats. Comparative analysis identified 178 significantly altered metabolites between the Blank and Model groups (Figure 1I). These differential serum metabolites were mainly enriched in pathways related to fructose metabolism, lipid metabolism, amino acid metabolism, purine metabolism, the tricarboxylic acid cycle, as well as pathways in anti-inflammatory and antioxidant, among others (Figure 1J). Notably, several key metabolites with substantial changes are implicated in disease pathogenesis: Lysophosphatidylcholines (LPCs) and lysophosphatidylethanolamines (LPEs) are associated with metabolic syndrome and cardiovascular diseases (CVD). Phosphatidylethanolamine (PE) is linked to β-amyloid (Aβ) deposition in neurodegenerative disorders like Alzheimer’s disease (AD) [29]. Glutamine supports immune cell proliferation, while leucine, by activating the mammalian target of rapamycin (mTOR) pathway, is closely associated with metabolic syndrome-related diseases and neurodegenerative disorders [30]. Glutamate functions as a neurotransmitter. Arachidonic acid derivatives play a role in the regulation of inflammation and oxidative stress. For example, 12(R)-Hydroxyeicosapentaenoic acid exhibits anti-inflammatory properties, and 8(S)-Hydroperoxyeicosatetraenoic acid, a lipoxygenase-derived metabolite, promotes neutrophil chemotaxis. Prostaglandin E2 levels show a positive correlation with inflammation. Primary bile acids, such as glycocholic acid, are involved in fat digestion. Beyond direct metabolic functions, certain metabolites like secondary bile acids (e.g., deoxycholic acid) also exhibit regulatory effects on the gut microbiota.

### 3.2. Metabolomics Analysis of Brain Tissue

**Figure 2:**
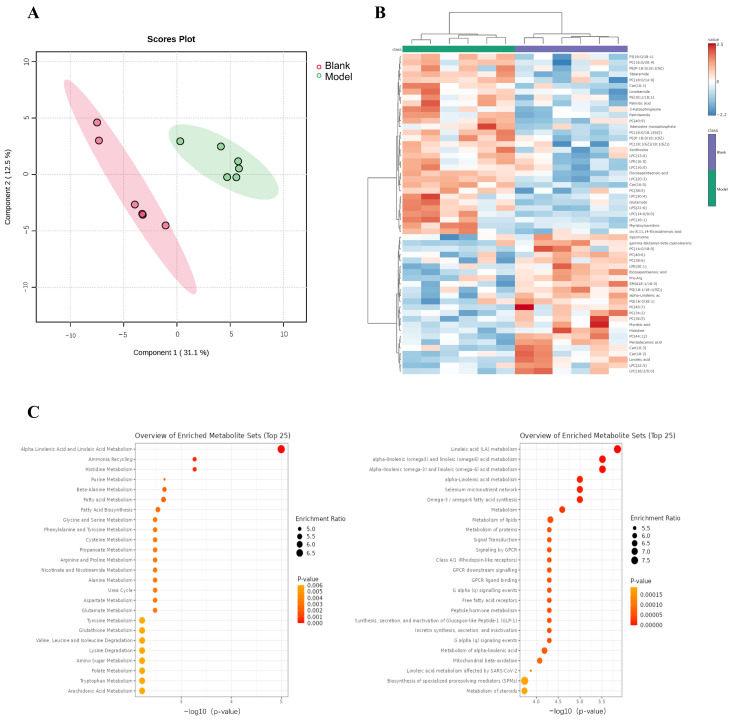
Metabolomics analysis of brain tissue. (**A**) PLS-DA score plot for the Blank group and Model group; (**B**) Heat map of brain differential metabolites between Blank and Model groups; (**C**) Biological process analysis of the differential metabolites between the Blank group and Model group.

Chronic poor dietary structure adversely affects brain function and increases the risk of CVD through metabolic disorders. Serum metabolomics results indicated that the rats exhibited metabolic disorders. Consequently, we examined the brain metabolome of these rats. The metabolomes of the brain tissue samples were analyzed by liquid chromatography-mass spectrometry in both positive and negative ion modes. The results of the PLS-DA dimensionality reduction analysis showed a clear separation between the Blank group and the Model group, indicating significant differences in brain tissue metabolite phenotypes between the two groups (Figure 2A). A total of 65 differential metabolites were identified when comparing the Blank group to the Model group (Figure 2B).

The metabolic pathway enrichment analysis of these differential metabolites is shown in Figure 2C. The differential metabolites were primarily enriched in pathways related to purine metabolism, fatty acid metabolism, amino acid metabolism, redox metabolism, folate metabolism, the urea cycle, the selenium micronutrient network, signal transduction metabolism, steroid metabolism, mitochondrial beta-oxidation, and peptide hormone metabolism. Among these metabolites, palmitic acid, a saturated fatty acid, has been implicated in both neurodegenerative diseases and metabolic syndrome. Elevated levels of palmitic acid in brain tissue induce endoplasmic reticulum stress and mitochondrial dysfunction, thereby promoting Aβ deposition, a key feature in the pathogenesis of AD [31]. Furthermore, high palmitic acid levels are associated with insulin resistance and obesity, potentially promoting neuroinflammation through activation of the toll-like receptor 4 pathway, which, in turn, can impair cognitive function [32]. Conversely, omega-3 fatty acids exhibit protective effects. As an omega-3 precursor, alpha-linolenic acid is converted to docosahexaenoic acid (DHA)/eicosapentaenoic acid (EPA). These metabolites regulate the PI3K/Akt pathway, ameliorating cerebral ischemia and reducing stroke risk. N-3 docosapentaenoic acid (DPA), a DHA precursor in the omega-3 conversion pathway, can reduce neuroinflammation by inhibiting the NF-κB pathway and may delay cognitive decline in AD patients, demonstrating anti-inflammatory and cognitive protective functions [33]. The mechanisms of neuroinflammation are also implicated in the effects of other metabolites. For example, LPC can promote neuroinflammation in AD and PD by activating microglia to release IL-6 and TNF-α. Several cohort studies have found that LPC is significantly elevated in the serum of patients with ischemic stroke, potentially exacerbating cerebral edema by disrupting the BBB [34]. PC analogs such as PC (18:1/18:1) show abnormal levels in obese brains. These alterations may impair insulin signaling through lipid raft modulation and correlate with metabolic disorders. Specifically, the change in lipid raft structure may disrupt membrane fluidity, thereby interfering with insulin receptor signaling [35]. Furthermore, PC (16:0/20:4) is associated with oxidative stress in epilepsy and cerebral ischemia; it increases lipid peroxidation via arachidonic acid metabolism. Excessive glutamate release leads to N-methyl-D-aspartic acid (NMDA) receptor overactivation, triggering calcium overload and neuronal death, a process associated with AD, epilepsy, and stroke [36]. Aberrant accumulation of glutamate-related tRNA fragments inhibits mitochondrial protein translation and exacerbates cognitive decline.

### 3.3. Proteomics Analysis of Brain Tissue

**Figure 3:**
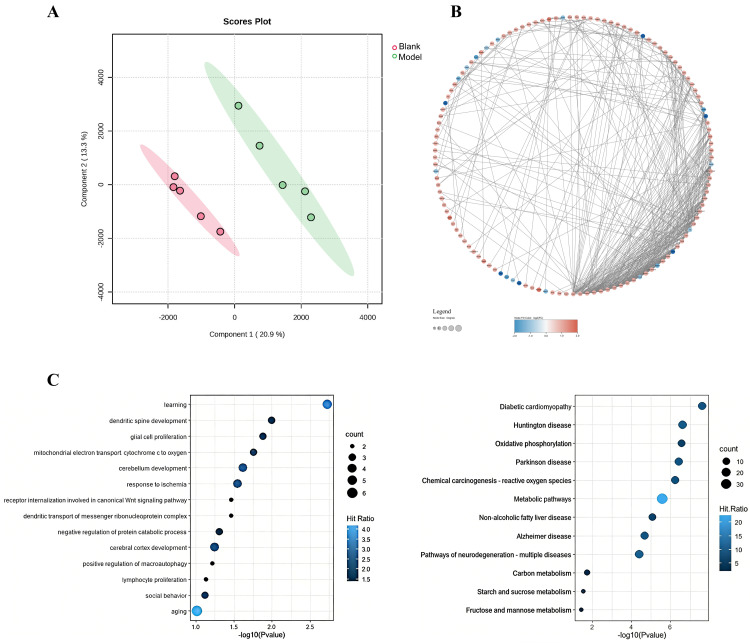
Proteomics analysis of brain tissue. (**A**) PLS-DA score plot for the Blank group and Model group, the red and green clusters correspond to the Blank and Model groups; (**B**) PPI network of the differential proteins between the Blank group and Model group, the size of the circle represents the magnitude of the degree value; (**C**) Biological process analysis of the differential proteins between the Blank group and Model group.

To comprehensively investigate protein alterations in the rat brain, we performed quantitative proteomic analysis. The protein distribution characteristics of the Blank and Model groups were evaluated using PLS-DA dimensionality reduction analysis (Figure 3A). The results showed a significant separation between the Blank and Model groups, indicating that a HSHFHFD resulted in proteomic alterations in the rat brain.

A comparison between the Blank and Model groups revealed 155 differentially expressed proteins, of which 134 were up-regulated and 21 down-regulated (Figure 3B). Protein–protein interaction (PPI) network analysis of these differentially expressed proteins revealed complex interaction patterns, suggesting their involvement in key biological processes.

The differentially expressed proteins were significantly enriched in pathways involving brain development, cognition and behavior, oxidative stress, immune regulation, and metabolism (Figure 3C). Among these, multiple proteins exhibit distinct pathological mechanisms in neurodegenerative diseases. Notably, presenilin 1, a core component of γ-secretase, mediates amyloid precursor protein cleavage generating amyloid-β (Aβ) and is associated with aberrant tau phosphorylation in AD [37]. Furthermore, VPS13A serves as a key mediator in the modulation of lysosomal-mitochondrial membrane contact and lipid transport and has been implicated in defective mitochondrial autophagy and PD [38]. Similarly, HK1 catalyzes glucose phosphorylation, regulates glycolysis, and is associated with ischemic brain injury. In parallel, CRP has been observed to co-localize with Aβ plaques in postmortem studies and promote microglial activation, which is associated with stroke and AD neuroinflammation. [39] As an ILK-binding protein, ILKAP regulates NF-κB signaling through the ILKAP-ILK axis, which exacerbates vascular permeability, thereby promoting inflammatory responses characterized by neutrophil infiltration.

We then performed a KEGG pathway analysis, and the results indicated that these proteins were associated with various pathological conditions, including Huntington’s disease (HD), PD, AD, non-alcoholic fatty liver disease (NAFLD), and diabetic cardiomyopathy. Notably, multiple neurodegenerative pathways exhibited significant correlations.

In summary, the proteomics results indicated that a HSHFHFD may affect normal brain development, neuronal cell proliferation, and neural signaling in rats. It may also generate oxidative stress and inflammation, and affect cognitive and behavioral functions.

### 3.4. Effects of HSHFHFD on the Expressions of BBB-Related Proteins in Rat Brain

Brain proteomics analysis revealed that a diet high in salt, fat, and fructose affects glial cell proliferation in the rat brain. Astrocytes, the most abundant glial cells, are critical for neuronal survival. Under pathological conditions, astrocytes undergo abnormal activation and release inflammatory factors that contribute to brain disease and BBB disruption, ultimately leading to neuroinflammation. GFAP is a well-established marker of astrocyte activation. BBB integrity is maintained by tight junction proteins, including ZO-1 and occludin, which serve as key indicators of BBB damage. To establish a link between the observed proteomic changes and these cellular markers, Western blot analysis was performed to quantify the protein expressions of GFAP, ZO-1, and Occludin.

**Figure 4:**
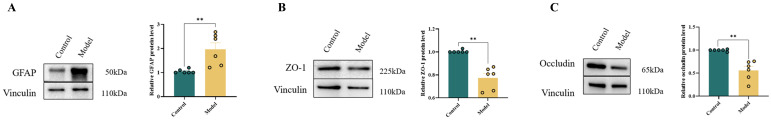
Expression of BBB-related proteins in rat brain. (**A**) GFAP; (**B**) ZO-1; (**C**) Occludin. Data are expressed as $\bar{x}$ ± s (*n* = 6); ** *p* < 0.01.

Quantitative analysis revealed that GFAP expression increased in Model group compared to blanks (*p* < 0.01, Figure 4A), while ZO-1 and Occludin expression were markedly lower in the Model group compared to the Blank group (*p* < 0.01, Figure 4B,C). These results suggest that HSHFHFD induces damage to the BBB, potentially through the activation of pro-inflammatory responses in astrocytes, leading to the degradation of tight junction protein.

### 3.5. Effects of HSHFHFD on Oxidative Stress and Inflammatory Levels in Rat Brain

**Figure 5:**
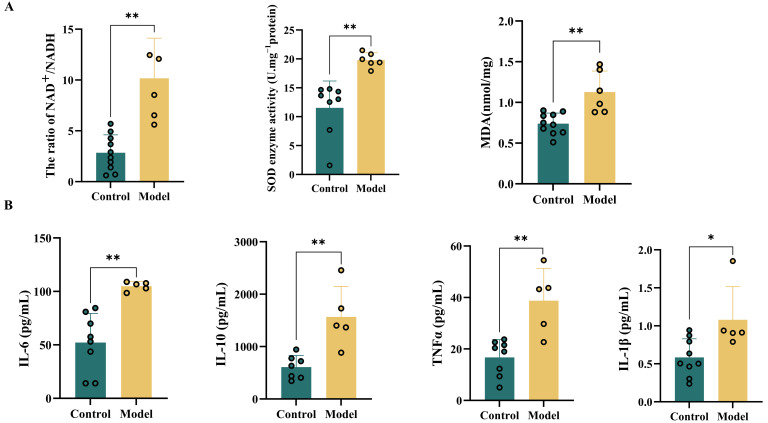
Levels of oxidative stress-related indicators and inflammatory factors in rat brain. (**A**) Levels of NAD^+^/NADH, SOD, and MDA in rat brain; (**B**) Levels of IL-6, IL-1, TNFα, and IL-1β in rat brain; Data are expressed as $\bar{x}$ ± s (*n* = 5~10); * *p* < 0.05; ** *p* < 0.01.

Brain proteomic analysis indicated that the HSHFHFD promoted oxidative stress and significantly affected inflammation-related processes in the rat brain. Damage to the BBB leading to cerebrovascular endothelial dysfunction has been implicated in the pathogenesis of oxidative stress. To assess the effect of the HSHFHFD on oxidative stress levels, we evaluated SOD activity and MDA concentration in rat brain tissue. As a key antioxidant enzyme, SOD catalyzes the disproportionation of superoxide radicals. MDA, the end product of lipid peroxidation, reflects the extent of oxidative damage. Compared with the Blank group, the level of NAD^+^/NADH, SOD, and MDA in the brain tissue of rats subjected to the HSHFHFD was significantly increased (*p* < 0.01, Figure 5A). These results suggest that the HSHFHFD may promote oxidative stress in the brains of rats.

Reactive astrocytes critically promote inflammatory responses. These secreted mediators subsequently stimulate microglia activation and immune cell recruitment, thereby inducing sustained neuroinflammatory responses [40]. To validate the observed inflammatory processes, inflammatory factors in the brain tissue of rats were determined. The results showed that elevated levels of IL-6, IL-1β, and TNF-α (Figure 5B) were observed in Model group. The concomitant increase in anti-inflammatory cytokine IL-10 may indicate compensatory regulatory mechanisms during neuroinflammation. Additionally, oxidative stress and inflammation are closely interconnected. Inflammatory cells release large amounts of ROS at sites of inflammation, exacerbating oxidative damage. Conversely, ROS and oxidative stress products further contribute to the inflammatory response.

### 3.6. Effects of HSHFHFD on CREB Signaling Pathway in Rat Brain

BDNF is critical for neuronal growth and differentiation. Increased BDNF expression inhibits oxidative stress and protects against neuronal death, playing vital roles in regulating learning and memory [41], cognitive function, and mood disorders. *p*-CREB promotes neuronal survival by upregulating downstream targets, including BDNF and the anti-apoptotic protein Bcl-2. Since Bcl-2 is a downstream target of CREB signaling and apoptosis is regulated by the balance between pro-apoptotic Bax and anti-apoptotic Bcl-2, we investigated the effect of HSHFHFD on the CREB signaling pathway in the rat brain.

**Figure 6:**
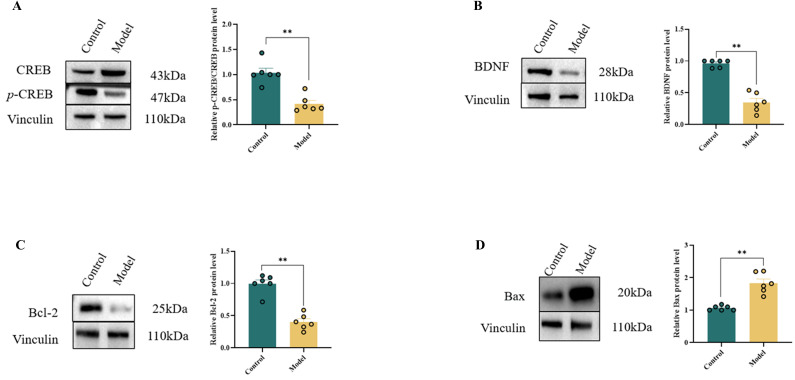
Expression of CREB pathway-related proteins in rat brain. (**A**) *p*-CREB/CREB; (**B**) BDNF; (**C**) Bcl-2; (**D**) Bax; Data are expressed as $\bar{x}$ ± s (*n* = 6); ** *p* < 0.01.

The *p*-CREB to CREB ratio, BDNF, and Bcl-2 protein levels in the brains of rats in the Model group were significantly lower than those in the Blank group (*p* < 0.01, Figure 6). Conversely, the expression level of the pro-apoptotic protein Bax was significantly increased (*p* < 0.01, Figure 6). This indicates that the CREB signaling pathway was down-regulated in the brains of rats in the Model group, leading to a decreased neuroprotective effect. This reduction in neuroprotection may be attributed to a decreased Bcl-2/Bax ratio, promoting mitochondrial pathway-mediated apoptosis.

### 3.7. Gut Microbiome Analysis

**Figure 7:**
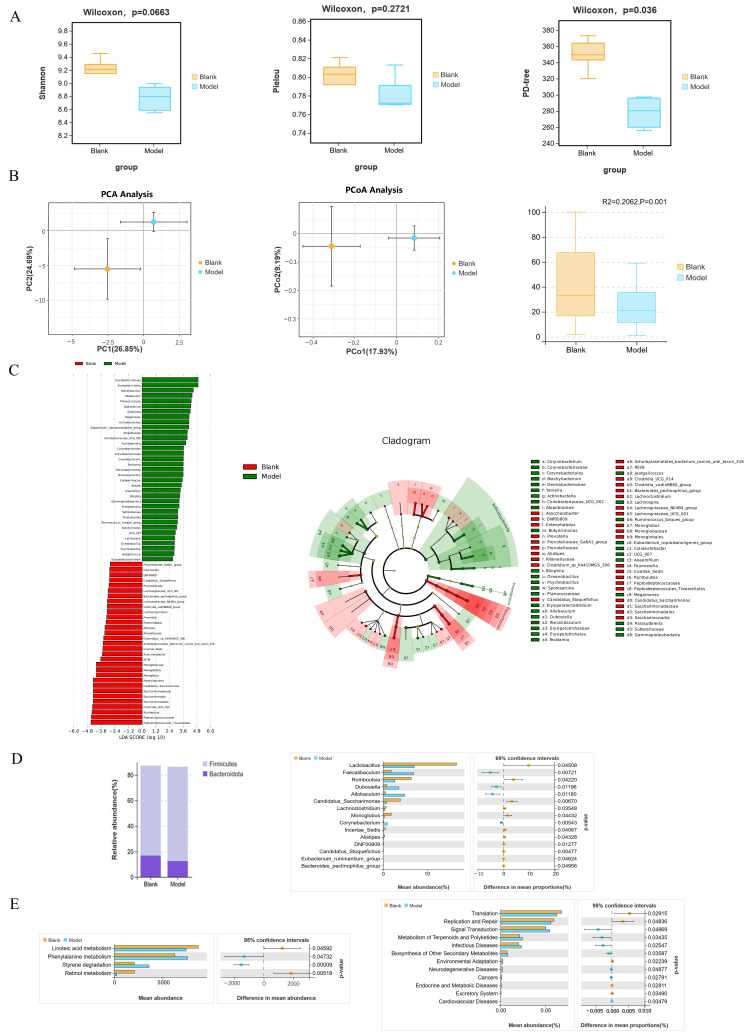
Gut Microbiome Analysis. (**A**) Effects of HSHFHFD on the alpha diversity of gut microbiota in rats (Shannon; Pielou; PD-tree); (**B**) PCA plots and PcoA analysis plots based on Bray–Curtis dissimilarity of the gut microbiota and Results of PERMANOVA test based on Bray–Curtis dissimilarity; (**C**) LEfSe analysis between the Blank group and Model group; (**D**) The difference in bacterial genera between the Blank group and Model group; (**E**) PICRUSt community function prediction, Differential pathways between the Blank group and Model group.

Diet exerts profound effects on the gut microbiota. We examined the gut microbiota in rats using 16S rRNA sequencing technology. Major α-diversity indices, including the Shannon-Wiener index, Pielou’s evenness index, and PD-tree were analyzed. Shannon indices reflected species richness and evenness, with higher values indicating greater diversity. Pielou indices primarily reflected species evenness, with larger values indicating a more even species distribution. PD-tree is a diversity index calculated based on the phylogenetic tree, considering the evolutionary relationships between species. Compared to the Blank group, the Model group showed a decrease in Shannon and Pielou indices (no significant difference), while the PD-tree index showed a significant difference between the two groups (Figure 7A).

Beta diversity reflects the overall composition of the microbiota. Principal component analysis (PCA) and principal coordinates analysis (PCoA) both demonstrate that the spatial distance between samples in the ordination plots reflects the similarity of their microbiome composition (Figure 7B). Under both algorithms, samples from all three groups showed significant separation, indicating differences in the gut microbiota composition among the groups. Notably, PERMANOVA testing confirmed a statistically significant difference between the Model and Blank groups (*p* = 0.001, FDR corrected) (Figure 7B), indicating that the modeling significantly altered the composition of the rat gut microbiota.

Differentially abundant bacterial taxa across phylum to genus levels were identified using LEfSe analysis. At the phylum level, the Blank and Model groups showed differences in 3 phyla (*Actinobacteriota*, *Proteobacteria*, *Patescibacteria*), 3 classes (*Actinobacteria, Gammaproteobacteria*, *Saccharimonadia*), and 8 orders (*Erysipelotrichales*, *Monoglobales*, *Saccharimonadales*, etc.). At the family level, 15 families showed differential abundance (*Monoglobaceae*, *Sutterellaceae*, *Atopobiaceae*, etc.), and at the genus level, 38 genera were differentially abundant (*Anaerofilum*, *Faecalibaculum*, etc.). A total of 35 genera were enriched in the Model group (Figure 7C). Welch’s *t*-test analysis revealed significant changes in the abundance of 15 bacterial genera at the genus level between the Blank and Model groups. Notably, the abundance of genera with putative beneficial roles, such as *Lactobacillus*, *Romboutsia*, *Candidatus-Saccharimonas*, and *Monoglobus*, was significantly reduced in the gut of rats in the Model group (Figure 7D). This decrease aligns with previous studies reporting the anti-inflammatory properties associated with these taxa [42].

To investigate the functional potential of the gut microbial communities, KEGG pathway enrichment analysis was performed based on OTU abundance profiles derived from fecal samples. Compared to the Blank group, the Model group exhibited altered abundance in several pathways. Welch’s *t*-test was used to analyze differential pathways between the groups. The KEGG pathway enrichment analysis results showed significant differences in several pathways between the Blank and Model groups (Figure 7E). These results suggest that the HSHFHFD altered the abundance of microbiota in the rat gut, resulting in an increase in the relative abundance of specific opportunistic pathogens and leading to changes in multiple functional pathways of the rat gut microbiota.

### 3.8. Multi-Omics Integration Analysis

**Figure 8:**
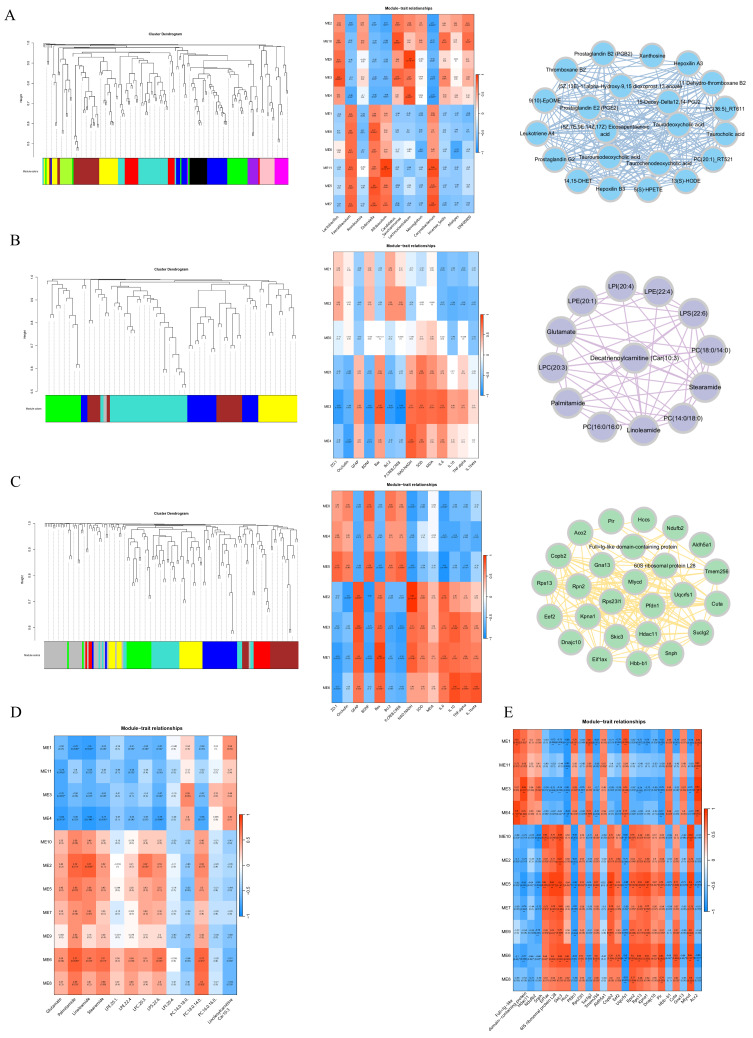
Multi-omics integration analysis through WGCNA. (**A**) Classification of Serum metabolome and Pearson correlation analysis; (**B**) Classification of brain metabolomes and Pearson correlation analysis; (**C**) Classification of brain proteomes and Pearson correlation analysis; (**D**) Correlation analysis of brain metabolome with serum metabolome; (**E**) Correlation analysis of brain proteome and serum metabolome. * *p* < 0.05; ** *p* < 0.01.

The integration of clustering data is crucial to elucidate the complex relationships between gut microbiota, proteins, and metabolites. To this end, we used the WGCNA R software (Version 4.1.3) package to integrate datasets from the serum metabolome, brain proteome, and brain metabolome. The clustering dendrogram for the serum metabolome is presented in Figure 8A. Modules of serum metabolites were then correlated with differential microbiota abundance to identify key metabolite modules. Our analysis revealed that the ME3 module showed the strongest correlation with differential microbiota (Figure 8A). It is important to clarify that while the ME3 module appears in metabolome analyses of both serum (Figure 8A) and brain (Figure 8B), their precise relationship requires further investigation, as they may represent distinct but related metabolic signatures. The gut microbiota most associated with ME3 is *Lactobacillus*, a probiotic that plays a key role in maintaining the micro-ecological balance of the gut. This balance is essential for maintaining gut health, inhibiting the growth of harmful pathogenic bacteria, strengthening the immune system against infection, and being closely associated with the prevention and treatment of a wide range of diseases, such as metabolic syndrome and cardiovascular disease [43]. WGCNA was then applied to the brain proteome and brain metabolome, with corresponding clustering dendrograms shown in Figure 8B,C, respectively.

To identify the modules with the greatest influence on the brain, proteomic and metabolomic data were correlated with experimental data on brain biochemical indicators. The ME3 module was most strongly correlated with the brain metabolome (Figure 8B), while the ME1 module correlated most strongly with the brain proteome (Figure 8C). The interrelationships within the ME3 module are depicted in Figure 8B, and the interactions within the ME1 module are shown in Figure 8C. Given that the blood pathway mediates gut microbiota-brain interactions, we correlated the brain proteome and metabolome with the serum metabolome. The correlation between the brain metabolome and the serum metabolome is illustrated in Figure 8D, and the correlation between the brain proteome and the serum metabolome is presented in Figure 8E.

Notably, our module-based analysis revealed two key molecular players: glutamate and ACC2/HDAC11. Association analysis of the brain metabolome indicated that glutamate was most strongly associated with the serum metabolome. As a key excitatory neurotransmitter, glutamate is critical for nerve signaling, neuronal growth, synapse formation, learning, and memory. Its strong association with neurodegenerative diseases, such as PD, highlights its importance in neurological health [36]. In the context of the brain proteome, ACC2 and HDAC11 emerged as the most strongly associated proteins. ACC2 is essential for fatty acid metabolism in the brain, supports energy metabolism in brain cells, and has neuroprotective effects. Furthermore, it regulates the expression of genes related to learning and memory, thereby enhancing synaptic plasticity and improving cognitive function. On the other hand, HDAC11 exhibits both neuroprotective and neurotoxic properties and affects neuronal survival, synaptogenesis, and synaptic plasticity through the modulation of histone acetylation and gene expression [44]. Specifically, HDAC11 is involved in adipogenesis, lipid metabolism, and energy expenditure, contributing to the regulation of brain energy metabolism, immune responses, and inflammatory processes. However, the precise mechanisms underlying these dual roles of HDAC11 require further investigation.

## 4. Summary and Discussion

Compelling evidence indicates that sustained consumption of diets rich in salt, saturated fat, and refined fructose contributes to nutritional imbalances. Additionally, such diets can impair the BBB integrity, thereby disrupting critical cerebral physiological functions. Consequently, these disruptions may increase susceptibility to diverse neurological disorders and cognitive dysfunction [45]. In this study, we modeled an 18-week unhealthy dietary pattern in SD rats using a high-salt, high-fat diet and 30% fructose water to investigate its effects on systemic health and brain damage.

Our findings demonstrate that a HSHFHFD increased blood glucose, blood pressure, and lipid levels in rats, concomitant with indicators suggestive of impaired liver function. It has been known that serum metabolites can directly contact and interact with the BBB, or enter the brain through the BBB, which in turn affects normal brain function. In addition, serum metabolites reflect the function and state of the brain. For instance, neurotransmitters and their metabolites in the serum, such as dopamine, serotonin, and glutamate, can indicate the neural activity and functional status of the brain [46]. Changes in dopamine levels are closely associated with neurological disorders like depression and PD [47]. Consequently, we conducted serum metabolomic analyses in HSHFHFD-fed rats. These analyses revealed significant perturbations in the serum metabolic phenotype of rats. These changes affected several metabolic pathways, including lipid metabolism, amino acid metabolism, purine metabolism, energy metabolism, and neural conduction. Among these pathways, arachidonic acid metabolism was the most prominently affected pathway. Arachidonic acid plays a crucial role in mediating cell signaling and inflammatory responses, regulating vasodilatation and platelet aggregation, and thus influencing blood pressure and coagulation. For example, the arachidonic acid derivative PEG2 exerts anti-inflammatory or pro-inflammatory effects by binding to various receptors and participating in pathways such as NF-κB and CREB [48]. Consequently, dysregulated arachidonic acid metabolism is closely related to CVD such as hypertension and atherosclerosis, metabolic diseases such as obesity, diabetes, and NAFLD [49], as well as neurodegenerative diseases like AD and PD [50].

Based on these results, we hypothesized that the model animals had metabolic disorders that could interfere with normal brain function. We therefore examined the brain metabolome and proteome of the rats. The results revealed marked perturbations in the brain metabolome, particularly in pathways related to fatty acid metabolism, amino acid metabolism, purine metabolism, hormone metabolism, oxidation-reduction, and signal transduction. Among these, the linoleic acid pathway was the most influential. Linoleic acid is an essential nutrient for brain development, promotes the development and activity of brain cells, and participates in the regulation of neurotransmission through its conversion to arachidonic acid, a precursor of neurotransmitters. In addition, linoleic acid has neuroprotective effects and can reduce neuroinflammation. For instance, studies demonstrate neuroprotective effects of linoleic acid in PD models, [51] while its metabolite *n*-6 DPA reduces neuroinflammation, inhibits microglial hyperactivation, and attenuates apoptosis in AD. At the same time, the brain proteome of the rats was significantly altered. Differentially expressed proteins were enriched in biological processes critical for learning, social behavior, glial cell proliferation, brain morphogenesis, oxidative phosphorylation, ROS metabolism, and energy metabolism. These proteins were strongly associated with neurodegenerative diseases such as HD, PD, and AD. This finding corroborates the results of the brain metabolome analysis.

To validate the proteomics results, we quantified the levels of relevant proteins, oxidative stress markers, and inflammatory factors in the rat brain using Western blotting and ELISA. The results revealed significant elevations of GFAP, impaired BBB tight junction structure (ZO-1, Occludin), oxidative stress markers (NAD^+^/NADH, SOD, MDA), and inflammatory factors (IL-6, IL-10, TNFα, IL-1β) in the brains of rats from the Model group. Conversely, the expression of neuroprotection-related pathways, including CREB, *p*-CREB, BDNF, and Bcl-2, was decreased. Specifically, BDNF can exert anti-inflammatory effects by activating downstream pathways such as PI3K/Akt, which suppresses the expression of pro-inflammatory factors (e.g., TNF-α, IL-1β, IL-6) and promotes that of the anti-inflammatory cytokine IL-10 [52]. The anti-apoptotic protein Bcl-2 directly protects neurons from cell death and, by maintaining cellular homeostasis, indirectly enhances neuronal resilience to inflammatory damage [53,54]. CREB is a key transcriptional regulator of BDNF and can also upregulate Bcl-2 expression. Furthermore, CREB has been shown to act in concert with the Nrf2/HO-1 pathway while antagonizing NF-κB signaling, thereby suppressing microglial-mediated neuroinflammation [55]. Consequently, the rats fed a HSHFHFD exhibit downregulated CREB expression, leading to reduced levels of BDNF and Bcl-2. Concurrently, HSHFHFD induces abnormal activation of astrocytes by elevating GFAP, leading to the release of inflammatory mediators. This process causes degradation of tight junction proteins, thereby inducing BBB damage. These results confirm the brain proteomics data and suggest that a HSHFHFD impairs the BBB of the rat brain, thereby increasing the risk of neurodegenerative diseases.

Dietary intake can directly influence the gut microbiota, and systemic circulation mediates gut–brain axis communication. Several studies have demonstrated a close relationship between gut microbiota dysbiosis and various non-communicable diseases, including CVD [56], obesity [57], metabolic diseases (e.g., diabetes) [58], and neurological diseases [59]. To explore the relationship between gut and brain, we examined the gut microbiota of rats using serum metabolomics and a battery of brain assays. The results revealed significant β-diversity shifts in the rat gut microbiota. Specifically, there was a decrease in the abundance of several gut-healthy bacteria, including *Lactobacillus*, *Romboutsia*, *Candidatus-Saccharimonas*, and *Monoglobus*. These changes were associated with pathways such as linoleic acid metabolism, bacterial replication and repair, nucleotide metabolism, and cell community prokaryotes. Notably, linoleic acid metabolism was the most significantly affected pathway. This finding aligns with previous observations that the conversion of linoleic acid to arachidonic acid is consistent with the brain metabolome and serum metabolomics results.

To investigate the systematic relationships between brain, gut microbiota, and serum changes, we performed multi-omics integration analysis using WGCNA. Our results suggest that *Lactobacillus* is the most highly associated gut microbiota. This microbiota is critical for maintaining the gut microecological balance, enhancing the immune system, and preventing various diseases, including cardiovascular problems and metabolic disorders such as metabolic syndrome [43]. Studies show that *Lactobacillus* can regulate adipose tissue differentiation and fatty acid oxidation via the PPARα/γ pathway [60]. In addition, *Lactobacillus* produces the inhibitory neurotransmitter gamma-aminobutyric acid, regulates the gene and protein expression of tight junction proteins, and modulates intestinal mucus secretion [61,62]. Consequently, they participate in mood regulation and cognitive processes while maintaining the intestinal epithelial barrier to prevent neuroinflammation caused by pro-inflammatory substances entering the circulation. Furthermore, *Lactobacillus* can convert linoleic acid to conjugated linoleic acid in the gut. Conjugated linoleic acid has anti-cancer, anti-inflammatory, and antioxidant properties, as well as modulates lipid metabolism by inhibiting lipoprotein lipase activity. It reduces TC and LDL levels in the blood while increasing HDL levels. In addition, conjugated linoleic acid improves vascular endothelial function, increasing the elasticity and dilatability of blood vessels, thereby helping to maintain normal blood pressure levels. This is closely related to cardiovascular and metabolic diseases such as atherosclerosis, type 2 diabetes mellitus, and NAFLD [63]. We hypothesize that a reduction in the abundance of *Lactobacillus* in the gut of rats leads to decreased conjugated linoleic acid content, resulting in lipid disorders, elevated blood pressure, and an increased risk of cardiovascular and metabolic disease in rats.

Among the brain metabolome modules, glutamate was the most relevant. This amino acid is involved in signaling between neurons in the brain, regulates neuroplasticity, and is associated with processes such as energy metabolism, learning, and memory. It is closely associated with neurodegenerative diseases. Arachidonic acid, a metabolite of conjugated linoleic acid, serves as a precursor to several active lipids, including prostaglandins and leukotrienes, which modulate glutamate release and receptor activity.

In the brain proteomic modules, ACC2 and HDAC11 emerged as the most highly correlated proteins. Both proteins are associated with essential brain functions such as learning and memory, neuroprotection, and energy metabolism. ACC2 activity is regulated by AMPK, and conjugated linoleic acid and its metabolites can indirectly regulate ACC2 activity and function by affecting AMPK activity [64]. In addition, prostaglandins and leukotrienes generated from arachidonic acid can regulate HDAC11 activity. In conclusion, these data suggest that a HSHFHFD disrupts the conjugated linoleic acid pathway in rats by reducing the abundance of *Lactobacillus* in the gut. This disruption may affect the BBB and neuroprotective function of the brain, increasing the risk of neurodegenerative disease in rats.

Furthermore, although SD rats are recognized as one of the standard strains in nutrition, metabolic research, and neuroscience, dietary formulations in animal studies represent high-intensity, sustained interventions designed to accelerate pathological processes for observation within experimental timeframes, whereas human diets are typically more complex and variable. Furthermore, although rat and human gut microbiomes share similarities, differences persist. Rats also possess distinct life cycles compared to humans. Consequently, while the findings from this study may possess certain applicability, their specific manifestation in other strains or species warrants further investigation. In addition, these associations require more in-depth research and more direct evidence for validation, such as the relationship between ACC2, HDAC11 and glutamate, longer-term effects on the rats. Nevertheless, this study provides important mechanistic insights into how poor dietary habits impact health, with particular relevance to the brain–gut axis.

## 5. Conclusions

Our study systematically investigated the effects of a HSHFHFD on the rat organism and brain and explored its potential implications for systemic diseases. By integrating proteomics, metabolomics, and microbiological techniques, we revealed a systemic relationship between changes in the rat gut microbiota and brain. The results suggest that HSHFHFD disrupts the gut microbiota, reducing *Lactobacillus* abundance. The reduction in *Lactobacillus* diminishes linoleic acid content and may further disrupt linoleic acid metabolism and lead to elevated blood pressure and dyslipidemia in rats. Concurrently, the disturbed gut microbiota appears to activate pro-inflammatory responses in astrocytes via the gut–brain axis, causing degradation of tight junction proteins. This induces BBB damage, compromising neuroprotective functions in the brain. Our findings suggest that this dietary pattern may potentially increase the risk of metabolic and neurodegenerative diseases in rats. However, these associations require more in-depth research and more direct evidence for validation. Despite these limitations, our study contributes to a deeper understanding of how dietary composition influences the gut–brain axis in health and disease states.

## Data Availability

The datasets presented in this study can be found in online repositories. The names of the repository/repositories and accession number(s) can be found below. The proteomics data have been deposited to the ProteomeXchange Consortium (https://proteomecentral.proteomexchange.org, accessed on 27 October 2025) via the iProX partner repository with the dataset identifier PXD069926. The data of serum metabolomics can be found at metabolights (https://www.ebi.ac.uk/metabolights/, accessed on 6 Novemeber 2025) at [https://www.ebi.ac.uk/metabolights/reviewer279991b8-ae3e-4f8a-bfbd-4267a6ef25e3, accessed on 6 Novmeber 2025]. The data of brain metabolomics can be found at metabolights (https://www.ebi.ac.uk/metabolights/, accessed on 6 Novemeber 2025) at [https://www.ebi.ac.uk/metabolights/reviewer755e1205-0423-49bb-9d69-6d5c0e4e100c, accessed on 6 Novemeber 2025]. The 16S rRNA sequencing data for rat gut microbiota can be found at subCRA054025 at GSA (https://ngdc.cncb.ac.cn/gsa/index.jsp, accessed on 6 Novmeber 2025).

## Abbreviations

HSHFHFD	High-salt, high-fat, and high-fructose diets
SD	Sprague-dawley
TG	Triglycerides
SCAFs	Short-chain fatty acids
TC	Total cholesterol
LDL	Low-density lipoprotein
GFAP	Glial fibrillary acidic protein
Bax	Bcl-2-associated X protein
ZO-1	Zonula occludens-1
CREB	Cyclic-AMP response binding protein
BDNF	Brain-derived neurotrophic factor
Bcl-2	B-cell lymphoma-2
IL-6	Interleukin-6
IL-10	Interleukin-10
IL-1β	Interleukin-1beta
TNFα	Tumor necrosis factor α
NAD	Nicotinamide adenine dinucleotide
NF-κB	Nuclear factor kappa-B
SOD	Superoxide dismutase
MDA	Malondialdehyde
BBB	Blood–brain barrier
KEGG	Kyoto encyclopedia of genes and genomes
AD	Alzheimer’s disease
LPS	Lipopolysaccharides
PD	Parkinson’s disease
WHO	World health organization
ELISA	Enzyme-linked immunosorbent assay
PLS-DA	Partial least squares-discriminant analysis
OPLS-DA	Orthogonal partial least squares-discriminant analysis
PPI	Protein–protein interaction network
PCR	Polymerase chain reaction
PCA	Principal component analysis
PCoA	Principal coordinates analysis
PERMANOVA	Permutational multivariate analysis of variance
LEfSe	Linear discriminant analysis effect size
PICRUSt	Phylogenetic investigation of communities by reconstruction of unobserved states
IS	Internal standard
QC	Quality control
ANOVA	Analysis of variance
ALB	Albumin
LPC	Lysophosphatidylcholines
PC	Phosphatidylcholines
LPE	Lysophosphatidylethanolamines
PE	Phosphatidylethanolamine
Aβ	β-amyloid
mTOR	Mammalian target of rapamycin
DHA	Docosahexaenoic acid
EPA	Eicosapentaenoic acid
DPA	Docosapentaenoic acid
NMDA	N-methyl-D-aspartic acid
VPS13A	Vacuolar protein sorting associated protein 13A
HK1	Hexokinase 1
CRP	C-reactive protein
ILK	Integrin-linked kinase
ILKAP	Integrin-linked kinase-associated phosphatase
HD	Huntington’s disease
NAFLD	Non-alcoholic fatty liver disease
ROS	Reactive oxygen species
OTU	Operational taxonomic units
WGCNA	Weighted gene co-expression network analysis
ACC2	Acetyl coenzyme A carboxylase 2
HDAC11	Histone deacetylase 11
HDL	High-density lipoprotein
AMPK	AMP-activated protein kinase
CVD	Cardiovascular diseases
PD	Phylogenetic diversity
